# Item selection, reliability and validity of the Shortness of Breath with Daily Activities (SOBDA) questionnaire: a new outcome measure for evaluating dyspnea in chronic obstructive pulmonary disease

**DOI:** 10.1186/1477-7525-11-196

**Published:** 2013-11-14

**Authors:** Teresa K Wilcox, Wen-Hung Chen, Kellee A Howard, Ingela Wiklund, Jean Brooks, Michael L Watkins, Charlotte E Cates, Maggie M Tabberer, Courtney Crim

**Affiliations:** 1 Evidera, 7101 Wisconsin Avenue, Suite 600, Bethesda, MD 20814, USA; 2 Formerly of Evidera, 7101 Wisconsin Avenue, Suite 600, Bethesda, MD 20814, USA; 3 GlaxoSmithKline, Stockley Park West, Uxbridge, Middlesex UB11 1BT, UK; 4 GlaxoSmithKline, 5 Moore Drive, PO Box 13398, Research Triangle Park, NC 27709, USA

**Keywords:** Chronic obstructive pulmonary disease, Dyspnea, Shortness of breath, Patient-reported outcome measure, Shortness of breath with daily activities questionnaire

## Abstract

**Background:**

Chronic obstructive pulmonary disease (COPD) is characterized by irreversible, progressive obstruction of lung airflow. Dyspnea (shortness of breath [SOB]) is the COPD symptom which most negatively impacts patients’ daily activities. To assess how SOB affects daily activities, 37 items were drafted through focus group discussions and cognitive interviews with COPD patients to develop a patient-reported outcome instrument: the Shortness of Breath with Daily Activities questionnaire (SOBDA). Psychometric analysis was conducted to reduce the number of items and evaluate the measurement properties of the final SOBDA.

**Methods:**

Prospective, observational study of 334 COPD patients, recruited from 24 pulmonology and internal medicine clinics in the United States. The 37-item SOBDA was administered to patients each evening for 28 days using an electronic diary. Patients answered every item and rated their level of SOB experienced that day during specific activities. Item selection was conducted by examining item characteristics, dimensionality, and Rasch model analysis results. The decision to delete an item was based on psychometric evidence, content validity, and expert clinical input. The final SOBDA instrument was evaluated for internal consistency, reproducibility, convergent validity, known-groups validity, and responsiveness.

**Results:**

Twenty-four items from the 37-item pool were removed following the item selection process: nine items were removed due to high item-to-item correlations; five due to floor effects; three due to infrequent activity; one due to gender bias; two due to low factor loadings; three due to unordered response options; and one due to expert’s discretion. Internal consistency and reproducibility of the final SOBDA were demonstrated by Cronbach Alpha = 0.87, and intra-class correlation coefficient = 0.91. Convergent validity was demonstrated by high correlation with the CRQ-SAS (0.60) and SGRQ-C (0.61). Known groups validity was demonstrated by significant difference between ratings of the mMRC and clinical global rating of severity. Evaluation of the ability to detect change was not performed owing to too few responders at the end of the study.

**Conclusions:**

Through the empirical item reduction process, 13 items were selected from the 37-item pool generated during qualitative development. The final 13-item SOBDA is a reliable and valid instrument for use in clinical trials.

## Background

Dyspnea, or shortness of breath (SOB), is a common and significant symptom of chronic obstructive pulmonary disease (COPD) and the most frequently cited reason for patients to seek medical attention [1]. The quality of life of patients with COPD is dramatically affected by the decline in functional status and physical activity associated with SOB [1-4].

Measurements of lung function such as forced expiratory volume in 1 second (FEV_1_) are the primary parameters used to monitor airflow limitation in COPD. However, lung function correlates poorly with the symptoms of COPD, including SOB, and may not reflect the symptomatic changes that are important to patients [1,5]. Monitoring the effect of treatment on SOB is important and can only be conducted from the patient’s perspective. Currently available instruments for assessing patient-reported symptoms of COPD, such as the Chronic Respiratory Questionnaire Self-Administered Standardised (CRQ-SAS) [6-8] and St. George’s Respiratory Questionnaire for COPD patients (SGRQ-C) [9-11], do not specifically focus on the concept of SOB associated with daily activity, being much broader in scope. In addition, there are no available instruments that support a SOB-specific claim for a medicinal product in the United States. As such, there is a need for a patient-reported outcome (PRO) measure that specifically addresses SOB associated with daily activities in COPD, and monitors change over time in response to treatment that can be used in clinical trials.

The Shortness of Breath with Daily Activities (SOBDA) questionnaire was specifically designed as an electronic patient-reported instrument to evaluate the impact of treatment on SOB in patients with COPD; its qualitative development has been previously described in detail [12]. Briefly, the development involved three distinct steps – focus groups, item development, and cognitive interviews – before items for the questionnaire were drafted. Focus groups of patients with a physician diagnosis of COPD were asked to describe their experiences of SOB during their daily activities. Based on this research and current literature reviews, a pool of items was drafted and subsequently discussed with instrument development experts and COPD clinical experts. Cognitive debriefing interviews that assessed the draft item pool were then conducted, and patient feedback was used to revise and update the items on the questionnaire.

The current study examined the measurement properties in order to reduce the number of items in the 37-item draft SOBDA questionnaire. The reliability, validity and responsiveness of the refined instrument were also assessed.

## Methods

### Study design

During the first stage of development [12], focus groups were held with COPD patients to explore concepts and themes related to SOB with daily activities. A total of seven groups (40 patients) were held to achieve concept saturation (no new descriptors or activities identified). After the initial questions (items) were drafted, cognitive debriefing interviews with 37 patients assessed this draft pool and provided feedback that was used to update and revise the items. Items included in the pool reflected both different levels of strenuousness and different body positions, as both have been demonstrated to be important in the subjective experience of dyspnea [3]. Clinicians, translational and lexibility experts were also consulted during the development process, and their input was incorporated into the questionnaire. The questionnaire was developed in line with the United States Food and Drug Administration (FDA) PRO guidance document stating that any PRO instrument must demonstrate evidence of reliability, validity, and ability to detect change [13]. The FDA (Division of Pulmonary, Allergy, and Rheumatology Products with consultation from Study Endpoints and Label Development Team, Office of New Drugs, Center for Drug Evaluation and Research) was also consulted during the development process.

The current stage of development, as reported here, was a prospective, observational study of patients with COPD (GlaxoSmithKline protocol 107364; Evidera A-4398) from 24 pulmonology and internal medicine clinics across the United States. Data were collected from January to September 2009. Patients were recruited with all levels of lung function to ensure that the final SOBDA was appropriate for use in all disease severities.

Two study visits were scheduled on Days 1 and 28. The study protocol and recruitment procedures met all Institutional Review Board and Health Insurance Portability and Accountability Act requirements, as well as applicable state and federal laws and regulations. Each participant provided written informed consent. The study was approved and monitored by Essex Institutional Review Board (Lebanon, New Jersey, USA).

### Study sample

Participants with all stages of COPD severity, as defined by the Global Initiative for Chronic Obstructive Pulmonary Disease (GOLD) guidelines [1], were recruited; see Table 1 for a full list of entry criteria. Maintenance medications were limited to stable prescriptions of inhaled long-acting beta-agonists, long-acting muscarinic antagonists, inhaled corticosteroids, long-acting beta-agonist/inhaled corticosteroid combination products, and/or Combivent®. Patients who were not previously prescribed maintenance medications were also included in the study population.

**Table 1:** Inclusion and exclusion criteria

**Key criteria**	
Inclusion	Exclusion
Current clinical diagnosis of COPD based on the GOLD 2008 guideline criteria [20]	
Ages 40–80 years	
Current or former smoker with smoking history of ≥ 10 pack-years	
Evidence of SOB confirmed by clinician mMRC dyspnea rating of ≥ 2 for GOLD spirometric classifications II–IV patients	
	Experienced an exacerbation in the 60 days preceding enrolment
	Concurrent diagnosis of asthma
	Known respiratory disorders other than COPD^a^
	Organic heart disease with resultant left ventricular failure and New York Heart Association class 2–4
	Known neuromuscular disease

^a^α-1 antitrypsin deficiency as the underlying cause of COPD, active tuberculosis, lung cancer, bronchiectasis, sarcoidosis, lung fibrosis, pulmonary hypertension and interstitial lung disease.

*COPD* Chronic obstructive pulmonary disease.

*mMRC* Modified Medical Research Council.

### Assessments and measures

Assessments completed by the clinician and patients are shown in Table 2. At Visit 1, patients completed a sociodemographic questionnaire, the CRQ-SAS (first administration) [6-8]; the SGRQ-C [9-11]; and the Modified Medical Research Council Dyspnea scale (mMRC; patient completed) [14]. Investigators completed a clinical questionnaire, mMRC scale, and Clinician Global Assessment of Dyspnea Severity (CGI-S). Study centers also conducted spirometry on all patients. At a predefined subset of clinical sites patients also completed a 6-Minute Walk Test (6-MWT) [15] and Borg rating scales (the Modified Borg Dyspnea Scale Participants [MBD] and the Borg Rating of Perceived Exertion Scale [RPE]) [16,17].

**Table 2:** Study assessments

**Criterion measures**^**a**^	**Study visit 1 (Day 1)**	**Daily on eDiary (Days 1–28)**	**Weekly on eDiary**	**Study visit 2 (Day 29 + 3)**
Participant-completed				
CRQ-SAS	X			X
SGRQ-C	X			X
mMRC dyspnea severity	X			X
Global Assessment of Shortness of Breath		X		
PGAC			X	
Clinician-completed				
mMRC dyspnea severity	X			X
CGI-S	X			
CGI-C				X
Respiratory impairment and exercise capacity				
Pre- and post-bronchodilator spirometry	X			X
6-MWT^b^	X			X
RPE (post-6-MWT)	X			X
MBD (pre- and post-6-MW)	X			X
Oxygen saturation (pre- and post-6-MWT)	X			X

^a^CRQ-SAS.

^b^The 6-MWT was completed by a subset of patients.

*6-MWT* 6-Minute Walk Test.

*CGI-C* Clinician Global Impression of Change.

*CGI-S* Clinician Global Assessment of Dyspnea Severity.

*CRQ-SAS* Chronic Respiratory Questionnaire Self-Administered Standardised.

*MBD* Modified Borg Dyspnea Scale.

*mMRC* Modified Medical Research Council Grading System.

*PGAC* Participant Global Assessment of Change.

*RPE* Rating of Perceived Exertion scale.

*SGRQ-C* St. George’s Respiratory Disease Questionnaire for COPD Patients.

Patients received training on Day 1 for the electronic Diary (eDiary), used to administer the 37-item draft SOBDA questionnaire each evening for 28 days. Items asked the patient to rate their level of SOB experienced during an activity. All eDiaries were returned at Visit 2 (Day 29–32). Additional information gathered each day via the eDiary included a global assessment of SOB, instances of healthcare provider contact, and rescue medication use. A personalized built-in alarm alerted patients to complete the eDiary 1 hour prior to their normal bed time. Patients transmitted data from the eDiary on a daily basis, and adherence was monitored via a dedicated web portal.

All patients completed the CRQ-SAS (follow-up administration), SGRQ-C, and mMRC (patient-completed form) and spirometry assessment at Visit 2. A second 6-MWT was also completed in the subset of sites. An exit evaluation of perceived patient burden associated with completing the eDiary on a daily basis was also completed. Investigators completed a follow-up clinical questionnaire, mMRC scale and Clinical Global Impression of Change (CGI-C). Unscheduled healthcare utilization and any medication changes between Visits 1 and 2 were also recorded for potential use in exploratory analyses. Patients with poorly controlled SOB often require additional healthcare attention and may change medications more frequently than patients with SOB that is well tolerated.

### Statistical analysis

All patients with ≥ 1 day of eDiary data were included in the analysis for item reduction, scoring and scaling assessments, and for the psychometric analysis. The eDiary did not allow individual items to be skipped, but it was possible to miss an entire daily entry. Unless otherwise specified, Day 1 data were used for the item psychometric analyses. Where Day 1 data were missing, the first diary entry completed was used. The frequency and possible reasons behind missing diary entries were examined by attempting to link days without a diary entry with data collected on the scheduled and unscheduled healthcare utilization and participant contact case report forms, as well as the participant’s report of healthcare provider contact on the preceding days.

### Item reduction

Item reduction was conducted by examining item characteristics, dimensionality, and the results of Rasch model analysis (see below for additional details, Rasch model analysis). The final decision to delete an item was based on the psychometric evidence, the content validity of the items, and expert clinical input. Day 1 diary data were used to assess the distributional characteristics of the individual SOBDA items; initial values were assigned to each category from 0 (‘I did not do the activity today’) to 5 (‘So severe that I did not do the activity today’). The mean, percentages of minimum and maximum responses (to determine floor and ceiling effects), item-total correlation, frequencies of item responses, and inter-item correlations were examined. An item was flagged for deletion if it had a floor (minimum response given by > 30% patients) or ceiling effect (maximum response given by > 50% patients). Additionally, items were flagged for deletion when the inter-item correlation was > 0.70, indicating duplication within the item pool; or the item-total correlation was < 0.20, indicating the question was measuring a concept different from the other questions in the item pool. Gender and age were examined to ensure that these factors did not contribute to how patients responded to the SOBDA items and prevent inherent biases in the final questionnaire; the cut-offs for these bias factors, gender and age, were set at 6% shared variance. In addition, a cut-off was set at 4% shared variance with the external criterion (clinician-rated dyspnea severity).

### Confirmatory factor analysis (CFA): assessment of dimensionality

CFA was conducted to determine whether the remaining items formed a uni-dimensional construct suitable for Rasch analysis. That is, all items which comprised the instrument measured the same construct (SOB with daily activities), and differed only by the levels of severity each of the items measured. Model fit was assessed using the comparative fit index (CFI), root mean square error of approximation (RMSEA) and standardized root mean square residual (SRMR). Good fit was defined as: > 0.90 CFI, and both RMSEA and SRMR < 0.05. Mplus software was used for factor analysis [18].

### Rasch model analysis

With the Rasch model, the probability of the patient responding to an item is modeled by a logistic function, with the assumption that a patient is more likely to respond to an item that reflects his/her condition [19]. Data were fit to a Rasch model with RUMM 2020 [20], and chi-square fit statistics were used to assess overall model and individual item fit. Item response thresholds and category probability curves were examined, and disordered response options were corrected by collapsing categories (e.g. ‘not at all’ and slightly’).

### Evaluation of measurement: reliability and validity

Internal reliability and validity were assessed with Cronbach’s Alpha and intra-class correlation (ICC) coefficients, respectively. The relationship between SOBDA scores and other measures (convergent validity) was assessed with Pearson’s correlation coefficient (6-MWT, SGRQ-C, CRQ-SAS, FEV_1_% predicted) or Spearman rank order correlation (mMRC score at Visit 1, MBD, RPE). The ability of the SOBDA to discriminate between clinically diverse groups (known groups validity) was assessed with analyses of variance in patients grouped by mMRC rating (clinician and patient), CGI-S rating, and GOLD stage. Construct validity was demonstrated through Pearson’s correlations with hypothesized variables (mMRC scores, CRQ-SAS domain scores, SGRQ-C).

## Results

**Figure 1:**
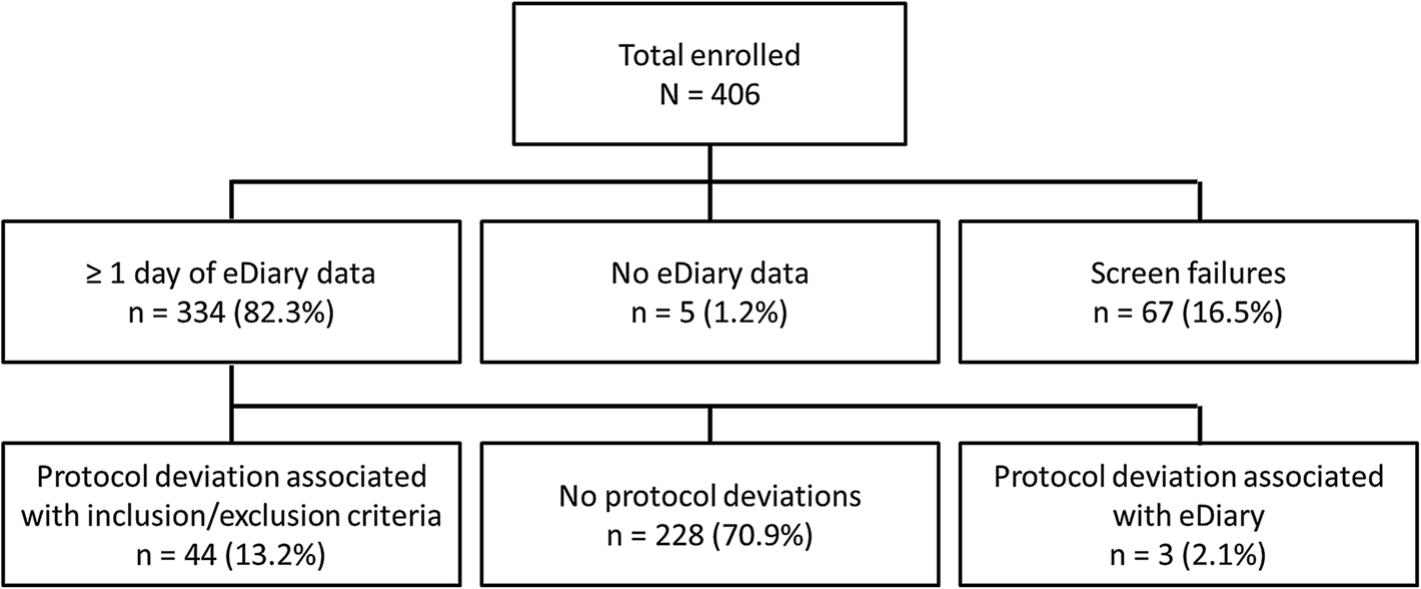
Patient flow.

Patient flow is shown in Figure 1, and patient demographic characteristics are summarized in Table 3. Of the 406 patients enrolled in the study, 67 patients were excluded from the analysis because of screen failures, including eight patients who were excluded due to Visit 1 protocol deviation. Five patients had no eDiary data. The final analysis population consisted of 334 patients. The mean age was 62.3 years and the majority of patients were White (76.9%). Approximately half of the population was male (52.1%) and current smokers (49.4%).

**Table 3:** Patient demographics

**Patient characteristic**	**Population for analysis (N = 334)**
Age, mean (SD)	62.3 (9.66)
Male gender, n (%)	174 (52.1)
Race/ethnicity, n (%)^a^	
American Indian or Native	8 (2.4)
Asian	39 (11.7)
Black or African American	15 (4.5)
Hispanic or Latino	18 (5.4)
Native Hawaiian or Other Pacific Islander	2 (0.6)
White	257 (76.9)
Other	3 (0.9)
Tobacco history, n (%)	
Current smokers	165 (49.4)
Former smokers^b^	169 (50.6)
Number of years since stopped smoking, mean (SD)	10.6 (9.15)

^a^Patients marked all that applied.

^b^Former smoker defined as not smoked within 6 months of Visit 1.

*SD* Standard deviation.

The mean time since COPD diagnosis was 7.4 years (Table 4) and, although patients with all levels of severity were included in the study, the majority of patients were classified by spirometry as GOLD Stage II (40.1%) or Stage III (34.1%). A similar distribution was also observed based on symptom severity (47.0% moderate; 30.2% severe) and symptoms as rated by clinicians completing a global impression of severity of SOB scale (48.5% moderate; 28.7% severe).

**Table 4:** Clinical characteristics

**Characteristics**	**Visit 1**
	**(N = 334)**
COPD diagnosis (years), mean (SD)	7.4 (6.6)
Chronic bronchitis, n (%)	90 (26.9)
FEV_1_% predicted (%), mean (SD)	51 (18.9)
COPD GOLD spirometric classification, n (%)^a,b^	
At risk	18 (5.4)
GOLD I (FEV_1_ ≥ 80% predicted)	19 (5.7)
GOLD II (50% ≤ FEV_1_ < 80% predicted)	134 (40.1)
GOLD III (30% ≤ FEV_1_ < 50% predicted)	114 (34.1)
GOLD IV (FEV_1_ < 30%)	48 (14.4)

^a^GOLD spirometric classification determined by Visit 1 post-bronchodilator spirometry assessment. ^b^Missing (n = 1).

*COPD* Chronic obstructive pulmonary disease.

*FEV*_*1*_ Forced expiratory volume in 1 second.

*SD* Standard deviation.

### Item analysis

**Figure 2:**
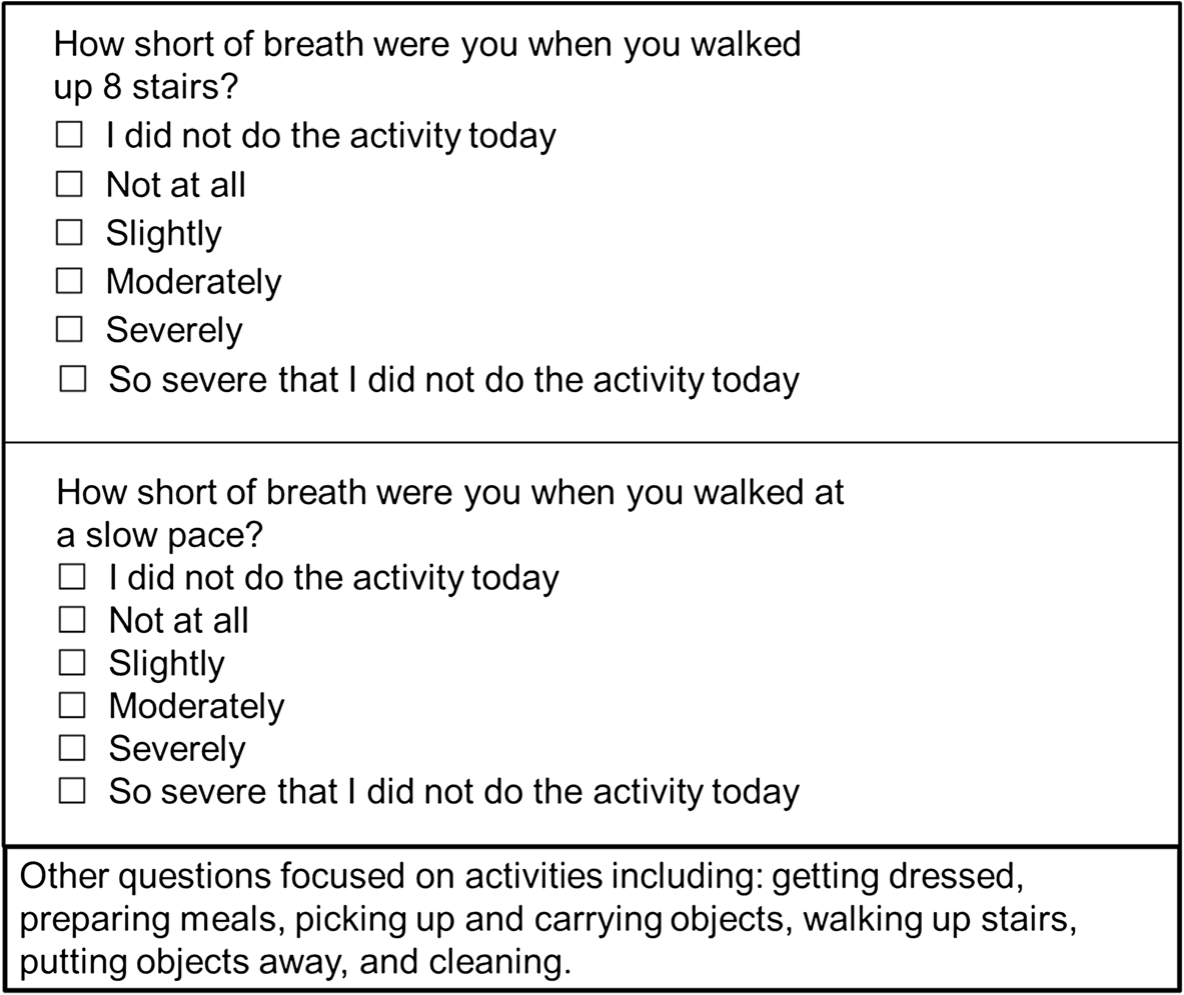
Rationale for item rejection or acceptance in the 37-item pool for the SOBDA questionnaire.

Of the 37 items, a total of 19 less relevant items were deleted from the original pool through item analysis; 10 of these were deleted due to high item-to-item correlations (duplication). The other nine items were deleted due to floor effects (five items), infrequently performed activity (three items), and gender bias (one item). The rationale for deletion or acceptance is shown in Figure 2. Additional items were later deleted from the pool based on CFA and Rasch model analysis.

### CFA: assessment of dimensionality

When CFA was conducted on the remaining 18 items in the reduced pool, the fit statistics (CFI = 0.837, RMSEA = 0.149, SRMR = 0.079) did not meet pre-defined criteria. Two items were identified for exclusion (‘make bed’ and ‘eating’) and were deleted based on low factor loading, leaving 16 items for further CFA. CFA on the remaining items did not substantially improve the fit statistics (CFI = 0.850, RMSEA = 0.150, SRMR = 0.080) but a general factor was strongly suggested by the high factor loadings. Recognizing that further removal of items would not improve the CFA fit statistics, Rasch model analysis was used to identify additional items for further exclusion.

### Rasch model analysis

Four items were removed from the 16-item pool based on Rasch model analysis (three items whose response options were not ordered and could not be collapsed; one item based on experts’ discretion). The one item (‘walked up 8 stairs’) deleted during the earlier item analysis due to its high correlation with two other items (‘walked up 3 stairs’ and ’walked up 8 stairs fast’) was re-instated due to its high relevance for measuring breathlessness in less severe patients and following recommendation by the expert clinical panel. A total of 13 items remained as a result of the item reduction process and formed a valid uni-dimensional scale, that is, all 13 items measured the same underlying construct of “SOB with daily activities”. The overall Rasch model fit had a chi-square of 88.48, degrees of freedom = 52, p = 0.001, and the person separation index was 0.86, showing good reliability. Chi-square statistics for individual item test-of-fit were not significant (p > 0.05) in 12 items; one item with p = 0.005.

### Scoring

When item reduction was complete, a finalized scoring system was created for the SOBDA questionnaire. The intent was to develop a scoring scheme that was consistent with the underlying framework and the empirical evidence of construct (i.e. items grouping around one main concept) derived through the analyses outlined above. The ‘did not do the activity today’ response was considered a missing score; other response categories were assigned ordinal scores based on the associated level of severity. Each daily SOBDA score was computed from the mean of the scores of the 13 items when there were at least seven scores not missing. The daily SOBDA score ranges from 1–4 with greater scores indicating more severe breathlessness with daily activities.

Daily scores frequently showed a wide variation in a patient’s activities from day to day, and therefore may not have accurately represented a patient’s overall experience of SOB related to activity. Mean scores for 2 or more days were therefore examined to determine which averaged values provided the most information. Weekly SOBDA scores were chosen as they accounted for the day-to-day variability and provided the best summary of overall experience of SOB . The final scoring algorithm therefore assigned each patient a weekly mean SOBDA score ranging from 1–4 with greater scores indicating more severe SOB with daily activities. The weekly score was calculated from the mean of the daily scores if data were available for at least 4 out of 7 days; the mean (SD) weekly score was 1.8 ± 0.6 (Week 1) and 1.8 ± 0.7 (Weeks 2–4), and the median weekly SOBDA score was 1.6 (range 1.0–4.0) across the 4 weeks of the study.

### Reliability and validity

SOBDA scores showed strong internal consistency and test-retest reliability. At Day 1, the internal consistency among the items (Cronbach’s Alpha) was 0.87; when individual constituent items were deleted values ranged from 0.85 to 0.87, indicating that no further item reduction was required. Assessment of weekly score test-retest reliability used the Participant Global Assessment of Change (PGAC) response of ‘no change’ to define stable patients. In these patients, Pearson’s correlation values were high and ICC coefficients were 0.91 for Week 1 to Week 2 and 0.87 for Week 1 to Week 4. In addition, differences in scores for Week 2 vs. Week 1 and Week 4 vs. Week 1 were small.

### Convergent validity

SOBDA weekly average scores showed appropriate convergent validity based on high correlation with other measures (Table 5). A strong relationship was observed between SOBDA, the dyspnea domain of the CRQ-SAS, and the activity domain of the SGRQ-C. The association between mean SOBDA scores during Week 1 and FEV_1_% predicted values from the Visit 1 spirometry was assessed. As expected, the relationship between lung function (FEV_1_% predicted) was relatively weak (r = –0.16) [1,5].

**Table 5:** **Correlations between SOBDA week 1 and FEV**_**1**_**% predicted, mMRC, 6-MWT, and COPD health status**

**SOBDA**	**n**	**Total score**
FEV_1_% predicted^a,b^	(n = 290)	–0.23*
mMRC^a^		
Patient^d^	(n = 292)	0.46*
Clinician^d^	(n = 292)	0.44*
6-MWT^a,c^		
Pre-MBD^d^	(n = 74)	0.62*
Post-MBD^d^	(n = 74)	0.58*
Borg RPE^d^	(n = 74)	0.63*
Distance^b^	(n = 74)	–0.46*
COPD health status^a^		
SGRQ-C total^d^	(n = 287)	0.73*
SGRQ-C symptom	(n = 290)	0.53*
SGRQ-C activity	(n = 290)	0.73*
SGRQ-C impact	(n = 288)	0.62*
CRQ-SAS dyspnea^d^	(n = 291)	–0.76*

^a^Measured as Visit 1.

^b^Pearson's correlation.

^c^6-MWTs were done at a subset of sites.

^d^Spearman's rank-order correlation.

*p < 0.0001.

*6-MWT* 6-Minute Walk Test.

*COPD* Chronic obstructive pulmonary disease.

*CRQ-SAS* Chronic Respiratory Questionnaire Self-Administered Standardised.

*FEV*_*1*_ Forced expiratory volume in 1 second.

*MBD* Modified Borg Dyspnea Scale.

*mMRC* Modified Medical Research Council.

*RPE* Rating of Perceived Exertion Scale.

*SGRQ-C* St. George’s Respiratory Disease Questionnaire for COPD Patients.

### Known-groups validity

SOBDA scores increased as patient and clinician mMRC ratings of severity increased, with more accurate discrimination observed for patient vs. clinician ratings. Analysis of Covariance (ANCOVA) showed that all pairwise comparisons of SOBDA scores between CGI-S ratings were significant (p < 0.05) with the exception of mild vs. moderate, and severe vs. very severe. Discrimination by GOLD stage was not observed, which was expected due to the low correlations between FEV_1_% predicted, SOBDA, and other PRO measures at Visit 1.

### Responsiveness

In this study there was no significant difference between responders (those who reported global improvement as ‘better’ or ‘much better’) and non-responders (those who reported no change, ‘worse’ or ‘much worse’); this is most likely due to the low number of patients reporting change (n = 4). Identification of a meaningful importance difference threshold was also explored, but the small sample size of responders prevented robust interpretation.

## Discussion

The development of item content and response options for the SOBDA questionnaire has been previously discussed [12]. This study was designed to appropriately reduce the number of items in the 37-item draft SOBDA questionnaire, and to examine the measurement properties: assessing the reliability, validity and responsiveness of the resultant instrument. The results demonstrate that the 13-item SOBDA is a reliable and valid tool for daily assessment of SOB during daily activities in patients with COPD for use in clinical research.

The internal consistency and reliability of the SOBDA total scores were demonstrated by the Cronbach Alpha of 0.87 and person separation index of 0.86 [21]. In addition, the SOBDA showed good reproducibility (ICC = 0.91) in patients reporting no change in their SOB (measured by the PGAC). Rasch and factor analyses further supported content validity, established uni-dimensionality, and confirmed that there were no gaps in measurement. Appropriate construct validity was also confirmed through Spearman rank order correlations with pre-specified variables including mMRC, MBD and RPE scores and Pearson’s correlations with 6-MWT distance, FEV_1_% predicted, CRQ-SAS domain scores and the SGRQ-C. Furthermore, a strong relationship was demonstrated between the SOBDA and the related dyspnea domain of the CRQ-SAS and activity domain of the SGRQ-C. These results provide evidence supporting the construct validity of the instrument, which is designed to measure SOB associated with activity. Although the relationship between SOBDA scores and mMRC was lower than expected, this association may have been affected by study inclusion criteria, which required patients to be symptomatic (mMRC ≥ 2) at screening. Although mMRC is often used for patient stratification within studies, it has previously been shown to be unresponsive to change and not sensitive enough to demonstrate a treatment effect in clinical trials [22,23]; this can be viewed as a limitation of this study. It should be noted, however, that no restriction on mMRC was used in recruiting patients with COPD for the qualitative stage of questionnaire development where the 37-items used in this study were developed [12]. The criteria used to recruit the patient population for this study are similar to those used in recruitment of clinical trials including the program where the SOBDA was initially used. These criteria are also consistent with FDA requirements for a clinical trial for symptom relief [13].

Both the FDA and the European Medicines Agency have recently emphasized the importance of reflected patient experience in clinical trials [13,24], as objective assessment may not accurately reflect symptomatic improvements; SOBDA can be utilized to provide these data. This further demonstrates the importance of developing a daily PRO instrument that specifically addresses SOB associated with daily activities in patients with COPD, which can monitor change over the course of treatment.

The low association between FEV_1_% predicted values and mean SOBDA scores was expected and is similar to results of other studies showing low levels of association between FEV_1_ and COPD symptoms, including SOB [1,5,25]. Results from the predefined subset of clinical sites which conducted exercise testing (patients completed the 6-MWT, MBD and RPE assessments) did not affect construct validity. These exercise assessments were not the primary endpoints of the study and did not influence the overall results.

Although responsiveness of the SOBDA was examined, almost all patients recruited to the study reported stable disease throughout the study period. The small sample of patients defined as responders (n = 4) prevented meaningful interpretation, which is another limitation of this study. However, an interventional study to define a responder threshold and confirm the responsiveness of the SOBDA was conducted and is reported separately [26]. That study expanded the current knowledge of the differences in scores among people with varying levels of severity of dyspnea and provided information on responsiveness.

It is important to note that the item pool evaluation used to achieve the 13-item questionnaire, and development of the questionnaire scoring were both conducted empirically to optimize the balance between patient burden in completing a daily questionnaire and measurement validity. To measure changes over time in symptoms such as SOB, which may vary on a daily basis, data collection using a diary is preferred as patients are more likely to recall their level of SOB while performing each activity accurately. This daily measurement of symptoms is also in line with guidance from the FDA for the development of a new PRO instrument [13]. Data collection methods where the daily entries are date and time stamped, such as the eDiary used in this study are also preferred.

The final SOBDA includes a number of different activities reflecting different levels of exertion and body positions which have been shown to impact a patient’s experience of SOB [3,12]. It was determined that the optimal method for scoring of the SOBDA was to use a weekly mean score (requiring data on at least 4 of the 7 days), based on the possibility that a patient may not conduct all daily activities each day. A weekly score is likely to maximize the available information provided by SOBDA without loss of responsiveness. In finalizing the scoring algorithm, it was important to ensure that patients were able to distinguish between the two options of ‘I did not do the activity today’ and ‘So severe that I did not do the activity today’. A response evaluation was therefore conducted, which included a re-examination of the qualitative research [12] and data from the current study, before finalising SOBDA scoring. Evaluation across the final 13 items over a 7-day period identified between 0–15 individuals (n = 334 responses) who endorsed both ‘I did not do the activity today’ and ‘So severe I did not do the activity today’ for a given item on different days suggesting that patients can distinguish between the two response options. However, implementation and scoring of this tool in a clinical trial setting would require further evaluation.

In respiratory clinical trials, patient-reported data historically have been collected through structured interviews and self-reported questionnaires, which may provide rich but not easily comparable data, and visual analogue or other numeric scales which may not adequately distinguish change with treatment [27,28]. While the patient-reported aspects of COPD have been assessed using currently available questionnaires, many instruments focus solely on symptom severity [6-11], and do not assess SOB with activity, which may be more meaningful to the patient. These tools are therefore not appropriate for drug development purposes where SOB with daily activity is the primary focus. For those instruments that include an assessment of SOB with activity, no consideration is given to posture, a critical component identified in the SOB endpoint rationale [3]. In addition, not all available assessments have been empirically validated to the standard required by the FDA guidance [13]. While including items related to activity, the CRQ-SAS [6-8] and the SGRQ [9-11] are instruments that measure multiple dimensions and are much broader in scope than SOB with activity, the target for the current SOBDA instrument.

## Conclusions

The SOBDA questionnaire was developed to address the need for a robust PRO instrument for use in clinical research to capture SOB associated with daily activities for patients with COPD. Through the use of quantitative evaluation and empirically-based item reduction, this prospective observational study successfully reduced the pool of 37 items generated from the qualitative phase of the questionnaire development to the final 13-item daily questionnaire. The validity and uni-dimensional nature of the final 13-item SOBDA questionnaire was also demonstrated; it showed good overall Rasch model fit and captured SOB along a single measurement scale, thus supporting the use of a total score to represent SOB with daily activities. The SOBDA questionnaire was found to be a reliable and valid instrument for measuring SOB with daily activity for COPD patients, and further analyses of the 13-item SOBDA questionnaire (responsiveness to change, response threshold, and minimal important difference) from a 6-week interventional study are awaited with interest.

## Abbreviations

6-MWT: 6-minute walk test; ANCOVA: Analysis of covariance; RPE: Borg rating of perceived exertion scale; COPD: Chronic obstructive pulmonary disease; CRQ-SAS: Chronic respiratory questionnaire self-administered standardised; CGI-C: Clinical global impression of change; CGI-S: Clinician global assessment of dyspnea severity; CFI: Comparative fit index; CFA: Confirmatory factor analysis; eDiary: Electronic diary; FEV1: Forced expiratory volume in 1 second; ICC: Intra-class correlation; MBD: Modified Borg dyspnea scale participants; mMRC: Modified Medical Research Council dyspnea scale; PGAC: Participant Global Assessment of Change; PRO: Patient-reported outcome; RMSEA: Root mean square error of approximation; SOB: Shortness of breath; SOBDA: Shortness of Breath with Daily Activities questionnaire; SGRQ-C: St. George’s Respiratory Questionnaire for COPD patients; SRMR: Standardized root mean square residual; FDA: United States Food and Drug Administration.

## Competing interests

Jean Brooks, Michael L Watkins, Maggie M Tabberer, and Courtney Crim are employees of, and own stock in, GlaxoSmithKline. Teresa K Wilcox, Ingela Wiklund, and Charlotte E Cates are employees of Evidera. Kellee A Howard and Wen-Hung Chen are former employees of Evidera. Funding to conduct the study, data analysis and interpretation, and generation of the study report was provided to Evidera by GlaxoSmithKline.

## Authors’ contributions

All authors meet the criteria for authorship set forth by the International Committee for Medical Journal Editors. All authors were involved in the data analysis and interpretation, drafting/revising publication for content, and approval of final version to be published. TKW, W-HC, KAH, IW, and MT were involved in the conception and design of study (protocol development and/or design advice), and TKW, KAH, and CEC were involved in the acquisition of data.
